# Human LYPD8 protein inhibits motility of flagellated bacteria

**DOI:** 10.1186/s41232-017-0056-3

**Published:** 2017-12-04

**Authors:** Chiao-Ching Hsu, Ryu Okumura, Kiyoshi Takeda

**Affiliations:** 1 Department of Microbiology and Immunology, Graduate School of Medicine, Osaka University, Osaka, 565-0871 Japan; 2 WPI Immunology Frontier Research Center, Osaka University, Osaka, 565-0871 Japan; 3 Core Research for Evolutional Science and Technology, Japan Agency for Medical Research and Development, Tokyo, 100-0004 Japan

**Keywords:** LYPD8, Glycosylation, *Pichia Pastoris*, Flagellated bacteria, Intestinal inflammation

## Abstract

**Background:**

We previously reported that the mouse Ly6/Plaur domain containing 8 (mLypd8), a GPI-anchored protein highly and selectively expressed on colonic epithelia, contributes to segregation of intestinal microbiota and intestinal epithelia and is critical for prevention of intestinal inflammation. In addition, it was found that human LYPD8 (hLYPD8) is expressed in the colonic epithelia and expression of hLYPD8 is reduced in some ulcerative colitis patients. However, the molecular characteristics and functions of hLYPD8 remain unclear. In this study, we generated the hLYPD8 protein and characterized its functions.

**Methods:**

To analyze the characteristics and functions of the hLYPD8 protein, recombinant FLAG-tagged hLYPD8 protein was generated by two kinds of protein expression systems: a mammalian cell expression system and a *Pichia pastoris* expression system. Recombinant hLYPD8 protein was analyzed by western blot analysis or deglycosylation assay. The effect of the protein on flagellated bacteria was examined by ELISA assay and motility assay using semi-agar plates.

**Results:**

hLYPD8 was a highly *N*-glycosylated GPI-anchored protein, like mLypd8. Moreover, recombinant hLYPD8 protein generated by the *Pichia pastoris* expression system using the SuperMan_5_ strain, which enabled production of a large number of proteins with human-like glycosylation, presented the high binding affinity and the motility inhibitory function to flagellated bacteria, such as *Proteus mirabilis*.

**Conclusions:**

These results demonstrated that hLYPD8 inhibits the motile activity of flagellated bacteria, many of which are involved in intestinal inflammation. The supplementation of recombinant hLYPD8 protein might be a novel therapeutic approach for intestinal inflammation of inflammatory bowel diseases.

## Background

Intestinal epithelial cells play important roles in the regulation of intestinal inflammation by generating several kinds of mucosal barriers, including mucus and antimicrobial peptides. These barriers contribute to the segregation of intestinal bacteria and the intestinal mucosa, which is indispensable for homeostatic maintenance of the gut where tremendous numbers of microorganisms live symbiotically [1–5]. Therefore, the dysfunction of mucosal barriers leads to the excessive immune response in the intestinal mucosa, which causes inflammatory bowel disease (IBD) represented by ulcerative colitis (UC) and Crohn’s disease (CD) [6]. Indeed, it has been reported that increased intestinal permeability and penetration of intestinal bacteria into the mucus layer due to barrier dysfunction are found in the intestine of IBD patients [7, 8].

In the colon, where tremendous numbers of microorganisms exist, intestinal microbiota and intestinal epithelial cells are clearly segregated by the thick mucus layer to maintain the symbiotic relationship between commensal microorganisms and the host [2, 3]. We previously demonstrated that the Ly6/Plaur domain containing 8 (Lypd8) expressed in intestinal epithelial cells promotes the segregation of intestinal bacteria and epithelial cells in the colon [9]. This molecule is an *N*-glycosylated GPI-anchored protein, and it binds to flagella and suppresses the motility of flagellated bacteria to prevent bacterial invasion of colonic mucosa. Furthermore, it was found that human LYPD8 (hLYPD8) is also expressed in the colonic epithelia, and the expression of LYPD8 is reduced in the colon of some UC patients. However, the molecular characteristics and functions of hLYPD8 remain unclear. We therefore analyzed the molecular characteristics and function of recombinant hLYPD8 protein generated by both mammalian cellexpression system and *Pichia pastoris* expression system.

## Methods

### Generation of HEK293T cells stably expressing mLypd8 and hLYPD8

Mouse Lypd8 (mLypd8) and human LYPD8 (hLYPD8) genes were amplified by PCR from mouse and human colonic cDNA. A FLAG-tagged sequence was inserted into the total mLypd8 and hLYPD8 coding sequence immediately downstream of the predicted N-terminal signal sequence. HEK293T cells obtained from ATCC were transfected with linearized pcDNA3.1 (+) vector (Invitrogen) inserted the sequence for FLAG-tagged mLypd8 or hLYPD8 using Lipofectamine2000 (Invitrogen). These cells were cultured in G418-containing medium. The surviving cells were stained with anti-FLAG M2 monoclonal antibody (cat F3165: Sigma-Aldrich) and Alexa Fluor 488 goat anti-mouse IgG antibody (cat A11001: Molecular Probes), and cells expressing FLAG-tagged mLypd8 or hLYPD8 were sorted using FACSAria (BD Biosciences).

### Purification of FLAG-tagged hLYPD8 proteins from HEK293T cells

Recombinant hLYPD8 protein was purified from HEK293T cells stably expressing FLAG-tagged hLYPD8 using FLAG M2 Purification Kit (Sigma-Aldrich). As a negative control, non-transfected cells were used.

### Assay for GPI cleaving activity

HEK293T cells stably expressing hLYPD8 (1 × 10^6^ cells) were rinsed twice with cold PBS and incubated with 0.5 ml of PBS containing 0.5 units of *Bacillus cereus* phosphatidylinositol-specific phospholipase C (PI-PLC, Molecular Probes) at 4 °C for 20 min. These cells were stained with anti-FLAG M2 mAb (Sigma-Aldrich) and Alexa Fluor 488 goat anti-mouse IgG (Invitrogen). The surface expression of hLYPD8 was analyzed using FACSCanto II (BD Biosciences).

### Deglycosylation assay

Recombinant hLYPD8 protein (1 μg) was incubated with PNGase F, sialidase A, and *O*-glycanase (Prozyme) at 37 °C for 3 h. Recombinant hLYPD8 protein treated with glycanase was separated with SDS-PAGE and transferred to polyvinylidene fluoride membranes (Millipore) that were incubated with horseradish peroxidase (HRP)-conjugated anti-FLAG M2 mAb (Sigma-Aldrich). Immunoreactivity was detected using Chemi-Lumi One (Nacalai Tesque).

### ELISA assay of LYPD8 binding to bacteria

Bacterial strains including *Escherichia coli* JCM 1649^T^, *Enterococcus gallinarum* JCM 8728^T^, *Bacteroides sartrii* JCM 17136^T^, and *Bifidobacterium breve* JCM 1192^T^ were obtained from the Japan Collection of Microorganisms. *Proteus mirabilis* was isolated from the colonic tissue of *Lypd8* ^−/−^ mice as described previously [9]. These bacteria were cultured at 37 °C for 16 h in an anaerobic chamber, fixed by 4% PFA, and stained with DAPI. The cell numbers were quantified by a confocal microscope. Each bacterium was centrifuged at 5000×*g* for 5 min, and the pellet was resuspended in 0.05 M sodium carbonate. The bacteria (1 × 10^7^ cells/ ml) were coated on 96-well plates (Corning) and incubated for 16 h at 4 °C. The plates were then washed with PBS and blocked with 1% BSA/PBS. After the plates were washed, the increasing concentrations of FLAG-tagged hLYPD8 protein diluted in PBS were added and incubated for 2 h at room temperature. Next, after the plates were washed, HRP-conjugated anti-FLAG M2 mAb (Sigma-Aldrich) in PBS was added and incubated for 1 h at room temperature. The plates were then washed, and 3,3′,5,5′-tetramethylbenzidine (TMB) substrate was added. Stop solution 1 M H_2_SO_4_ was then added, and the plates were read at 450 nm with a spectrometer.

### Pull-down assay for LYPD8 binding to flagella

Bacterial suspension of *P. mirabilis* or *E. coli* JCM 1649^T^ in PBS was shaken 300 times per min for 60 min to remove flagella from bacterial bodies. Bacterial bodies were pelleted by centrifuging at 4000×*g* for 20 min. The supernatants were ultracentrifuged at 80,000×*g* for 60 min to obtain flagella. Bacterial bodies or flagella were mixed with the solution of FLAG-tagged recombinant LYPD8 (10 ng/μl) and incubated for 3 h at 4 °C. After incubation, the bacterial bodies or flagella were pelleted down by centrifugation or ultracentrifugation, respectively. Then, the supernatant was collected and the pellet was resuspended in PBS. The supernatant and pellet suspension were separated with SDS-PAGE and transferred to polyvinylidene fluoride membranes (Millipore) that were incubated with HRP-conjugated anti-FLAG M2 mAb (Sigma-Aldrich). Immunoreactivity was detected using Chemi-Lumi One (Nacalai Tesque).

### Generation of *Pichia pastoris* expressing glycosylated hLYPD8 protein

The host strain *Pichia pastoris* SuperMan_5_ strain (*HIS* ^*+*^) and the expression vector pJAZ-aMF vector were purchased from Biogrammatics Inc. (Carlsbad, USA). The FLAG-tagged LYPD8 gene without C-terminal signal sequence was amplified by PCR and ligated into pJAZ-aMF vector.

*P. pastoris* SuperMan_5_ competent cells were prepared by a high-efficiency transformation condition suggested by Wu et al. [10]. Adequate amounts of constructed plasmids were digested with *Pme* I or *Sac* I to linear form and purified, then added to *P. pastoris* competent cells and well mixed. The mixed sample was added into a 2-mm gap cuvette (Bio-Rad, USA) and placed on ice for 5 min. The linearized DNA fragment was transformed into *P. pastoris* competent cells by the electroporation condition of 1.5 kV, 25 μF, and 200 Ω. Then, 1 ml of 1 M sorbitol was added into the sample and incubated at 30 °C for 1 h. At last, the sample was poured evenly into YPDSZ (1% yeast extract, 2% peptone, 2% dextrose, 1 M sorbitol, 100 μg/mL zeocin) agar plates then cultured at 30 °C for 2–3 days. Fifty colonies were selected to the new YPDZ agar plates with different zeocin gradients (100, 300, and 500 μg/ml) for testing the zeocin resistance and cultured at 30 °C for 1–2 days. Few transformants with better zeocin resistance were selected for further analysis.

### Purification of FLAG-tagged hLYPD8 proteins generated by a *Pichia pastoris* expression system

The selected transformants were inoculated in 4 ml of YPDZ for 24 h as a seed culture, then transferred into 500 ml BMGY (1% yeast extract, 2% peptone, pH 6.0 100 mM potassium phosphate, 1% glycerol, 1.34% yeast nitrogen base, and 4 × 10^−5^% biotin) and cultured at 30 °C, 200 rpm for 24 h. The medium of each transformant was replaced with 100 ml of BMMY (1% yeast extract, 2% peptone, pH 6.0 100 mM potassium phosphate, 1.34% yeast nitrogen base, 2% methanol, and 4 × 10^−5^% biotin). The transformants were then cultured for 48 h at 20 °C, 200 rpm. At the 24-h time point, 2% methanol (2 ml) was added to the culture. Soluble FLAG-tagged hLYPD8 protein was purified from 100 ml of the supernatant by column chromatography according to the FLAG M2 Purification Kit (Sigma-Aldrich).

### Motility assay of *Proteus mirabilis* and *Escherichia coli* in semisolid agar

HEK293T cells (5 × 10^7^ cells) with or without FLAG-tagged hLYPD8 were lysed with 1 ml of CelLytic M Cell Lysis Reagent (Sigma-Aldrich). After centrifuging at 13,000×*g* for 10 min, the supernatant was incubated with 150 μl of anti-FLAG M2 affinity gel (Sigma-Aldrich) for 3 h. The resin was centrifuged and washed with Tris-buffered saline three times. The resin containing FLAG-tagged hLYPD8 was mixed with 2 ml of lysogeny broth (LB) medium containing 0.3% agar. The final concentration of hLYPD8 protein in the LB agar was estimated by SDS-PAGE and was approximately 1.5 μg/ml. In the case of usage of soluble hLYPD8 protein generated by *P. pastoris*, 0.5 ml of recombinant soluble hLYPD8 protein was mixed with 1.5 ml of the LB medium containing 0.3% agar. The final concentration of hLYPD8 protein in the LB agar was estimated by SDS-PAGE and BCA assay, and it was approximately 50 μg/ml.

*P. mirabilis* or *E. coli* was cultured in the LB medium at 37 °C until OD_600_ was 0.6. The semisolid LB agar (0.3%) containing hLYPD8 protein was centrally inoculated with 1 μl of bacterial culture and incubated at 37 °C. Motility was assessed by examining circular migration. The radius of circles formed by bacterial migration was measured at 4 h after the bacterial inoculation.

### ELISA assay for LYPD8 binding to flagella

Several 96-well plates (Corning) were coated with 100 μg/ml flagella of *P. mirabilis* or 1% BSA/PBS for 16 h at 4 °C. The plates were then washed, and increasing concentrations of FLAG-tagged hLYPD8 protein diluted in 1% BSA/PBS were added and incubated for 2 h at room temperature. The plates were then washed, and HRP-conjugated anti-FLAG M2 mAb (Sigma-Aldrich) diluted in 1% BSA/PBS was added and incubated for 2 h at room temperature. The plates were then washed, and TMB substrate was added. The plates were then read at 450 nm with a spectrometer.

### Statistical analysis

Data are presented as mean ± s.d., as indicated in the figure legends. Differences between control and experimental groups were evaluated using a two-tailed unpaired Student’s *t* test. A *P* value of < 0.05 was considered significant. No statistical methods were used to predetermine sample size. No sample was excluded from the analysis.

## Results

### Molecular characteristics of the hLYPD8 protein

**Fig. 1:**
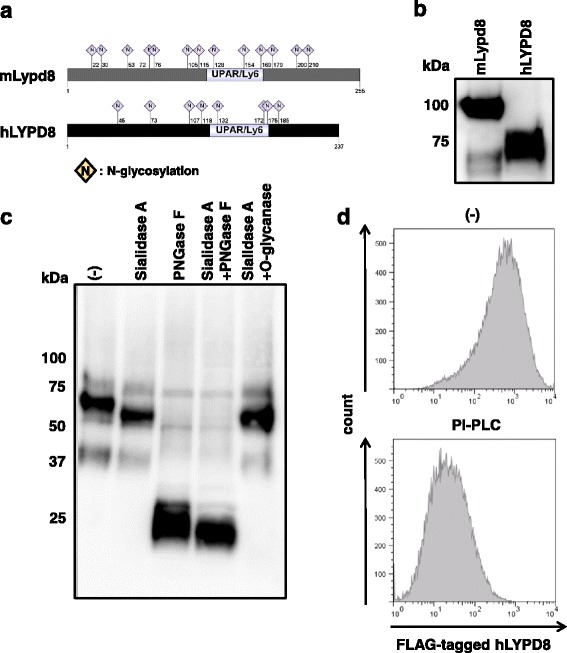
Molecular characteristics of the hLYPD8 protein. **a** Predicted glycosylation sites of the mLypd8 and hLYPD8 proteins. Glycosylation sites were referred to the UniProt database, and the picture was drawn by a group-based predictive system (IBS 1.0.2). **b** Immunoblotting for whole cell lysates of HEK293T cells expressing mLypd8 and hLYPD8. **c** Immunoblotting for recombinant hLYPD8 protein untreated or treated with sialidase A, PNGase F, or *O*-glycanase. **d** Flow cytometric analysis of HEK239T cells expressing FLAG-tagged LYPD8 before (upper) and after (lower) treatment with PI-PLC

LYPD8 is broadly conserved in mammalian species, including human, mouse, and rat. We previously showed that mouse Lypd8 (mLypd8) is a highly *N*-glycosylated protein. The hLYPD8 protein also contains eight glycosylation sites predicted by the amino acid sequence (Fig. 1a). We first purified recombinant hLYPD8 protein from HEK293T cells stably expressing FLAG-tagged hLYPD8 and analyzed the protein by SDS-PAGE and western blot analysis. Although the expected molecular weight from amino acid length is about 20 kDa, a band for recombinant hLYPD8 protein was observed at about the 75-kDa position (Fig. 1b). In addition, the treatment with peptide-N-glycosidase F (PNGase F), which cleaves *N*-oligosaccharide chains from glycoproteins, substantially reduced the molecular weight of the hLYPD8 protein. In contrast, treatment with sialidase A and *O*-glycanase did not markedly change the molecular weight of the hLYPD8 protein (Fig. 1c). These data indicate that hLYPD8 is a highly *N*-glycosylated protein, like mLypd8. We next treated HEK293T cells expressing FLAG-tagged hLYPD8 with phosphatidylinositol-phospholipase C (PI-PLC), which cleaves GPI-anchored proteins from the cell membrane, and analyzed hLYPD8 surface expression by flow cytometry. Treatment of PI-PLC severely reduced hLYPD8 surface expression, indicating that hLYPD8 is a GPI-anchored protein, similar to mLypd8 (Fig. 1d).

### Functions of the hLYPD8 protein generated by a mammalian cell expression system

**Fig. 2:**
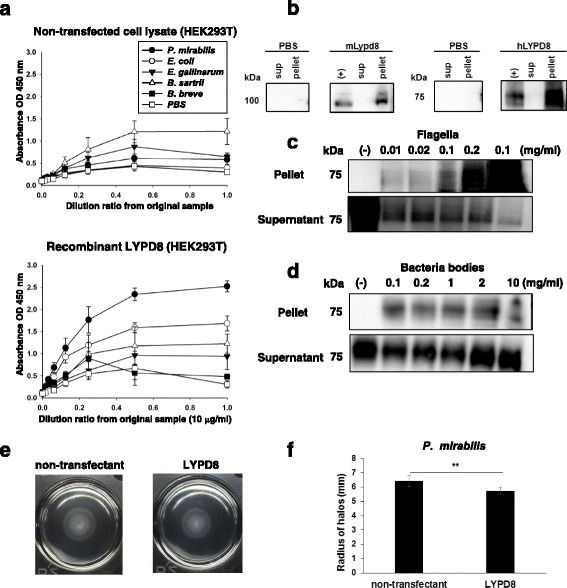
Function of the hLYPD8 protein generated by a mammalian cell expression system. **a** ELISA assay for hLYPD8 binding to intestinal bacteria. The quantified bacteria were coated onto microtiter plates and incubated with a dilution series of purified FLAG-tagged hLYPD8 (lower) or control sample (purified sample of non-transfected HEK293T cell lysate) (upper). **b**–**d** Immunoblot analysis with the anti-FLAG antibody for the supernatant and pellet of the mixture of FLAG-tagged mLypd8 or hLYPD8 and flagella from *P. mirabilis* (**b**) and the mixture of hLYPD8 and a gradient dose of flagella (**c**) or bacterial bodies (**d**) from *P. mirabilis*. **e, f** Motility of *P. mirabilis* in semisolid agar with the hLYPD8 protein or a control sample (a purified sample of non-transfected HEK293T cell lysate). Representative photos were shown (**e**). The radii of motility halos were measured at 4 h (**f**). Data are mean ± s.d. (*n* = 6 per group). **p < 0.01

The molecular characteristics of hLYPD8 are similar to those of mLypd8. Therefore, we next investigated the functions of recombinant hLYPD8 protein generated by a mammalian cell expression system. In the previous study, the mLypd8 protein was found to preferentially bind to flagellated bacteria, such as *E. coli* and *P. mirabilis*. Hence, we examined whether the hLYPD8 protein produced in mammalian cells bind to various kinds of intestinal bacteria including *P. mirabilis*, *E. coli*, *E. gallinarum*, *B. sartrii*, and *B. breve* by an ELISA assay (Fig. 2a). The hLYPD8 protein preferentially bound to *E. coli* and *P. mirabilis*, both of which are flagellated bacteria. Next, isolated flagella and bacterial bodies from cultured *P. mirabilis* were incubated with the hLYPD8 protein and then pelleted down by centrifuging. The hLYPD8 protein and the flagella were coprecipitated (Fig. 2b). After incubation with increasing flagella concentration, the amount of the hLYPD8 protein in the supernatants was decreased, whereas it was increased in the pellets (Fig. 2c). The incubation with bacterial bodies did not alter the amount of hLYPD8 in the supernatants (Fig. 2d). These data indicate that the hLYPD8 protein binds to flagella of *P. mirabilis*. Therefore, we tested whether recombinant hLYPD8 protein inhibits the motile activity of *P. mirabilis* in a semi-agar plate. The swarm circle diameter was reduced in the semi-agar plate containing the hLYPD8 protein (Fig. 2e, f), indicating that the hLYPD8 protein inhibits the swarming motility of *P. mirabilis*, as does the mLypd8 protein.

### Generation of the hLYPD8 protein by a *Pichia pastoris* expression system

**Fig. 3:**
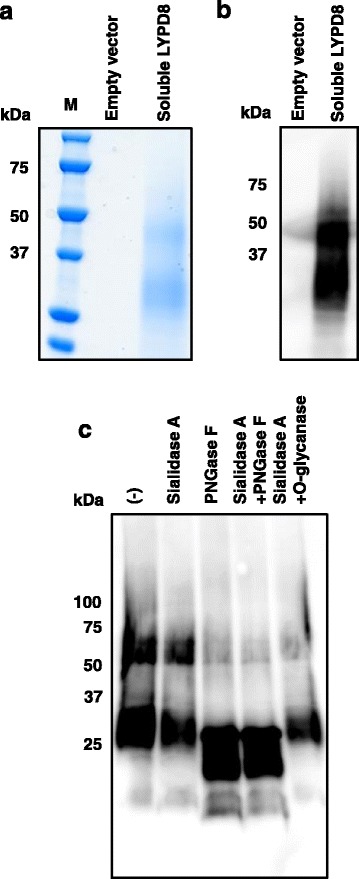
Molecular characteristics of the hLYPD8 protein by a *Pichia pastoris* expression system. **a, b** Protein samples purified from the culture media of *Pichia pastoris* transfected with empty vector or soluble hLYPD8 expression vector were separated by SDS-PAGE followed by Coomassie staining (**a**) and anti-FLAG immunoblotting (**b**). **c** Immunoblotting for recombinant soluble hLYPD8 protein untreated or treated with sialidase A, PNGase F, or *O*-glycanase

To extensively explore the functions of hLYPD8, a high quantity and quality of recombinant hLYPD8 protein are required. Considering the limitation of the protein-expressing ability of mammalian cells, we established another recombinant protein expression system for hLYPD8 by using the *Pichia* GlycoSwitch system [11]. This system uses the *Pichia pastoris* SuperMan_5_ strain, a methylotrophic yeast, which has strong promoters to drive the expression of a foreign gene. Moreover, it is a genetically modified strain that enable the production of large amounts of human-like glycosylated proteins with an easier technique and lower cost than other eukaryotic systems. Our previous mouse study revealed that the soluble form of mLypd8, shed from intestinal epithelial surface, acts on intestinal bacteria. Therefore, we constructed a soluble hLYPD8 expression vector by inserting a FLAG-tagged hLYPD8 sequence without a C-terminal signal sequence to the original *Pichia* GlycoSwitch vector. Recombinant hLYPD8 protein purified from the culture supernatant of *P. pastoris* transformed with the hLYPD8 expression vector was analyzed by SDS-PAGE and western blot analysis (Fig. 3a, b). Two bands for soluble hLYPD8 protein were observed at around the 50 and 30-kDa positions. These analyses suggested that the purified soluble hLYPD8 protein solution included about 50 kDa molecular weight of completely glycosylated hLYPD8 protein and 30 kDa molecular weight of partially glycosylated hLYPD8 protein. We next tested whether soluble hLYPD8 protein generated from *P. pastoris* was *N*-glycosylated, like GPI-anchored hLYPD8 protein generated by the mammalian cell expression system. The treatment of PNGaseF reduced the molecular weight of soluble hLYPD8 protein, indicating that soluble hLYPD8 protein generated by the *Pichia* GlycoSwitch system is *N*-glycosylated (Fig. 3c).

### Functions of the hLYPD8 protein generated by a *Pichia pastoris* expression system

**Fig. 4:**
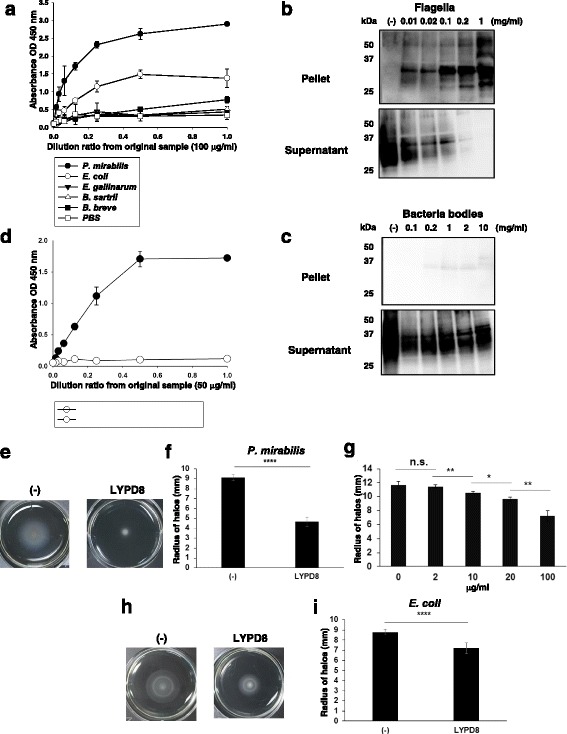
Function of the hLYPD8 protein generated by a *Pichia pastoris* expression system. **a** ELISA assay for binding of soluble hLYPD8 protein from *P. pastoris* to the indicated species of intestinal bacteria. **b, c** Immunoblot analysis with anti-FLAG antibody for the supernatant and pellet of the mixture of soluble hLYPD8 protein and a gradient dose of flagella (**b**) or bacterial bodies (**c**) from *P. mirabilis.* **d** ELISA assay of LYPD8 binding to flagella of *P. mirabilis*. **e, f** Motility of *P. mirabilis* in semisolid agar with or without soluble hLYPD8 protein. Representative photos are shown (**e**). The radii of motility halos were measured at 4 h (**f**). Data are mean ± s.d. (*n* = 6 per group). **g** Motility of *P. mirabilis* in semisolid agar with a gradient concentration of soluble hLYPD8 protein. The radii of motility halos were measured at 4 h. Data are mean ± s.d. (*n* = 6 per group). **h, i** Motility of *E. coli* in semisolid agar with or without soluble hLYPD8 protein. Representative photos are shown (**h**). The radii of motility halos were measured at 4 h (**i**). Data are mean ± s.d. (*n* = 6 per group). *p < 0.05, **p < 0.01, ****p < 0.001. n.s. not significant

We next examined the functions of soluble hLYPD8 protein generated by the *P. pastoris* expression system. We analyzed the binding of soluble hLYPD8 protein to several kinds of intestinal bacteria by an ELISA assay. We found that soluble hLYPD8 protein showed the stronger binding to *P. mirabilis* and *E. coli*, compared with the other non-flagellated bacteria (Fig. 4a). We then analyzed whether soluble hLYPD8 protein binds to flagella of *P. mirabilis* by the ELISA and pull-down assays (Fig. 4b–d). The results of both binding assays revealed that soluble hLYPD8 protein binds to flagella of *P. mirabilis* with high affinity. Subsequently, we analyzed the effect on the motile activity of bacteria. Soluble hLYDP8 protein remarkably inhibited the swarming ability of *P. mirabilis* in a dose-dependent manner (Fig. 4e–g). Moreover, soluble hLYPD8 protein also inhibited the motility of *E. coli* (Fig. 4h, i). These data clearly demonstrated that highly glycosylated soluble hLYPD8 protein generated by the *P. pastoris* expression system suppresses the motility of flagellated bacteria by binding to the flagella.

## Discussion

We previously demonstrated that mLypd8 contributes to the segregation of intestinal bacteria and intestinal epithelial cells through inhibiting bacterial invasion of colonic epithelia [9]. In this study, we characterized the hLYPD8 protein. We found that the hLYPD8 protein generated by the mammalian cell expression system was a highly *N*-glycosylated GPI-anchored protein. In addition, the ELISA binding assay showed that the hLYPD8 protein preferentially bound to flagellated bacteria such as *P. mirabilis* and *E. coli*. The motility assay revealed that the hLYPD8 protein inhibited the motility of flagellated bacteria in a semi-agar plate. These results indicate that hLYPD8 has the same characteristics and functions as mLypd8.

Glycans of secretory or membrane proteins such as mucins, produced by intestinal epithelial cells, are critical for the maintenance of mucosal barriers [12, 13]. In the case of mLypd8, PNGase F treatment reduced the inhibitory function of mLypd8 on intestinal epithelial cells, suggesting that *N*-glycans are important in the function of Lypd8 [9]. *N*-glycosylation varies across species and types of cells. The glycosylation in mammalian cells is the most suitable for mammalian glycosylated proteins to function appropriately. However, the ability of protein expression in mammalian cells is limited. Therefore, we generated soluble hLYPD8 protein by using a *Pichia* GlycoSwitch system, which makes it possible to generate large amounts of humanized-glycosylated protein at a relatively low cost. Both recombinant hLYPD8 proteins generated by mammalian cells and *P. pastoris* were highly *N*-glycosylated, which suggests that both recombinant proteins include similar glycans. However, the molecular weight of both proteins was different, indicating that there seems to exist some dissimilarity in the glycosylation pattern between both proteins. Although the *Pichia* strain we used for heterologous protein expression has been engineered to generate more human-like product by mutating the yeast *OCH1* gene which encodes α-1,6-mannosyltransferase resulting in high mannose-type of *N*-glycan and introducing heterologous enzyme activities [11], there remain many differences in glycosylation pathway between yeasts and mammalian cells [14]. Hence, the glycosylation pattern of *Pichia pastoris* generated protein is similar but not completely same as that of the protein produced by mammalian cells, and it is important to analyze the glycosylation pattern of both recombinant hLYPD8 proteins using mass spectrometry or lectin array analysis in the future. Despite the different glycosylation patterns, soluble hLYPD8 protein from *P. pastoris* showed a strong inhibitory effect for bacterial motility of flagellated bacteria. This result suggests that the difference in glycosylation between the mammalian cell expression system and the *Pichia* GlycoSwitch system does not affect the protein conformation influencing the protein activity. In addition, the purified soluble hLYPD8 protein solution included two forms of hLYPD8 proteins, fully or not fully glycosylated. Although soluble hLYPD8 protein contained a large amount of the immature form of the protein, it represented the inhibitory function against flagellated bacteria. Therefore, soluble hLYPD8 protein from *P. pastoris* can be used for further analyses of hLYPD8 protein function.

## Conclusion

Collectively, we found that hLYPD8 has the same characteristics and functions as mLypd8, and we successfully generated the functional highly glycosylated hLYPD8 protein by using the *P. pastoris* expression system, which enables generation of large amounts of recombinant protein. However, it remains unclear how the LYPD8 protein inhibits the motility of flagellated bacteria. Therefore, it is crucial to analyze the mechanism by which the LYPD8 protein binds to the flagella and inhibits the flagellar motile activity. Nonetheless, there is no convincing therapeutic approach targeting the dysfunction of mucosal barriers in IBD patients. Thus, a challenge exists in examining whether the supplementation of the hLYPD8 protein can reduce the severity of intestinal inflammation by regulating the motile activity of flagellated bacteria, many of which are related to intestinal inflammation [15, 16].

## Abbreviations

CD: Crohn’s disease
HRP: Horseradish peroxidase
IBD: Inflammatory bowel diseases
Lypd8: Ly6/Plaur domain containing 8
PI-PLC: Phosphatidylinositol-specific phospholipase C
PNGase F: Peptide-N-Glycosidase
TMB: 3, 3′,5,5′-Tetramethylbenzidine
UC: Ulcerative colitis
